# A Review of Computer-Aided Diagnostic Algorithms for Cervical Neoplasia and an Assessment of Their Applicability to Female Genital Schistosomiasis

**DOI:** 10.1016/j.mcpdig.2023.04.007

**Published:** 2023-06-13

**Authors:** Emily Jin, J. Alison Noble, Mireille Gomes

**Affiliations:** a Department of Computer Science, University of Oxford, United Kingdom; b Institute of Biomedical Engineering, Department of Engineering Science, University of Oxford, United Kingdom; c Global Health Institute of Merck, Ares Trading S.A., an affiliate of Merck KGaA, Darmstadt, Germany

## Abstract

Female genital schistosomiasis (FGS) affects an estimated 56 million women and girls in Africa. Nevertheless, this neglected tropical disease remains largely understudied and underdiagnosed. In this literature review, we examine the effectiveness of published computer-aided diagnostic (CAD) algorithms for cervical cancer that use colposcopy images and assess their applicability to the design of an automated image diagnostic algorithm for FGS. We searched 2 databases (Embase and MEDLINE) from database inception to June 10, 2022. We identified 393 studies, of which 13 were relevant for FGS diagnosis. These 13 studies were analyzed for their key image analysis model components and compared with the features that would be beneficial in an FGS diagnostic image analysis system.

**Article Highlights**

• In this literature review, we identified 13 published computer-aided diagnostic (CAD) algorithms for cervical cancer as potentially relevant for adaptation to automated image diagnosis for female genital schistosomiasis (FGS).
• Important features for selection of CAD algorithms for FGS include high sensitivity performance, ability to identify symptomatic areas, and efficient model design that can function in low-resource settings.
• Improvements are needed in the following areas to accelerate the development of FGS CAD algorithms: standardization of data collection and ground truth labels, data set quality and availability, consistency in reporting of published algorithms, and publicly available codes.

Schistosomiasis is a parasitic disease that affects ∼230 million people, most of whom live in low-income and middle-income countries (LMICs).^1^ Currently, schistosomiasis is the second most prevalent parasitic disease after malaria, and it is one of the most devastating tropical diseases with public health burden and economic effect.^2^ It is endemic to ∼51 countries globally, with millions more people at risk owing to inadequate hygiene and contact with infected water.^1^

An estimated 56 million women and girls in Africa experience female genital schistosomiasis (FGS),^3^ which accounts for almost a quarter of the global schistosomiasis cases. FGS is primarily caused by an infection by the Schistosoma (*Schistosoma haematobium*) parasitic worm that is found in contaminated bodies of water in LMICs.^1^ Infection is the result of direct contact with contaminated fresh water. Women will often come into direct skin contact with this infested water while performing daily chores and activities.

*S. haematobium* parasites penetrate the skin and lay eggs within the human host body, potentially affecting organ function. In the case of FGS, untreated infection can cause reproductive organ damage, most commonly in the cervix, which may lead to infertility and, if a woman is pregnant, spontaneous abortion, premature birth, and rarely maternal death.^4^ FGS shares several similar symptoms to sexually transmitted infections (STIs), such as irregular bleeding, pain during sexual intercourse, pelvic pain, and infertility, which can contribute to its misdiagnosis.^4^ There are also a number of studies linking FGS to cervical neoplasia and increased likelihood of contracting human immunodeficiency virus and human papillomavirus (HPV).^5^, ^6^, ^7^ Because of the effects on fertility and shared symptomology with STIs, women who experience FGS can also face substantial social ostracization, leading to serious mental health issues.^2^

Currently, the most accurate diagnostic method for FGS is to perform a biopsy to look for the presence of the *S. haematobium* eggs, but such a procedure is impractical in many of the areas where FGS is endemic.^2^ Not only are biopsies expensive, but they are also extremely intrusive and will often leave an open wound on the cervix. Because human immunodeficiency virus is also prevalent in many of the LMICs, where FGS is endemic, such an intrusive procedure is not a realistic option.^2^^,^^8^

Instead, the current clinical standard for FGS diagnosis is to perform a visual colposcopy examination (Figure 1), where clinicians inspect the cervix and vaginal wall for characteristic lesions.^2^ During a colposcopy examination, a healthcare worker will insert a speculum into the vaginal canal to get an unobstructed view of the cervix. With the speculum holding the vaginal walls apart, the healthcare worker can shine a light on the cervix and visually inspect it for any lesions or any irregularities in surface appearance.

**Figure 1:**
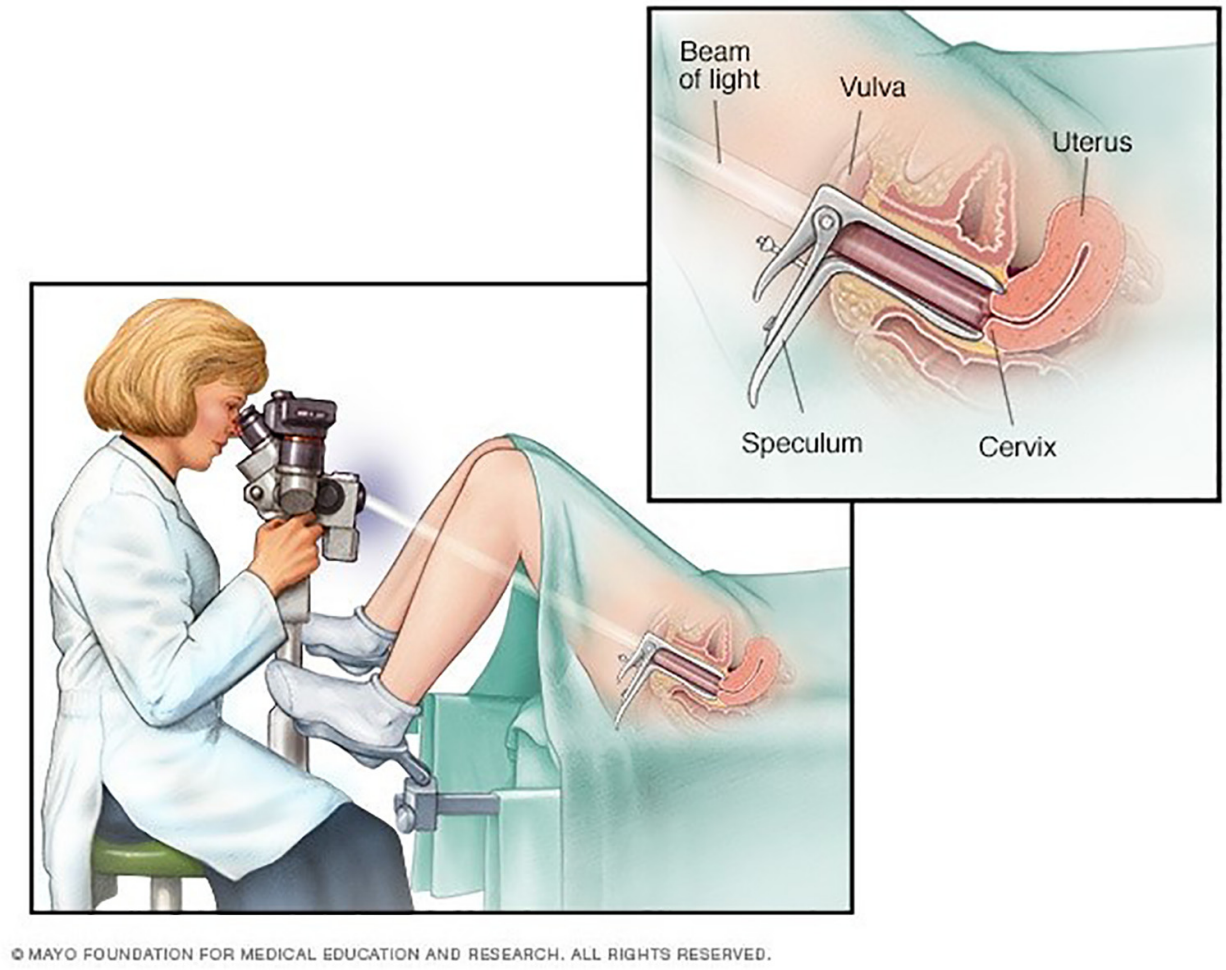
Diagram of a colposcopy examination. From the Mayo foundation for medical and educational research,^9^ with permission.

In the case of FGS, these abnormalities present as grainy sandy patches, homogenous yellow sandy patches, abnormal blood vessels, or rubbery papules (Figure 2).^4^ However, these lesions can often be misdiagnosed as other STIs given their similar appearances and the lack of education on FGS provided to healthcare workers in the field.^4^ Different experts who have had different training may also disagree in their diagnoses. An automated computational approach would remove a number of these biases and could help standardize the diagnostic processes for FGS. In addition, smartphone-based automated approaches may provide cost-effective solutions for use in the resource limited endemic settings, with minimal digital infrastructure needs and training requirements for healthcare workers.

**Figure 2:**
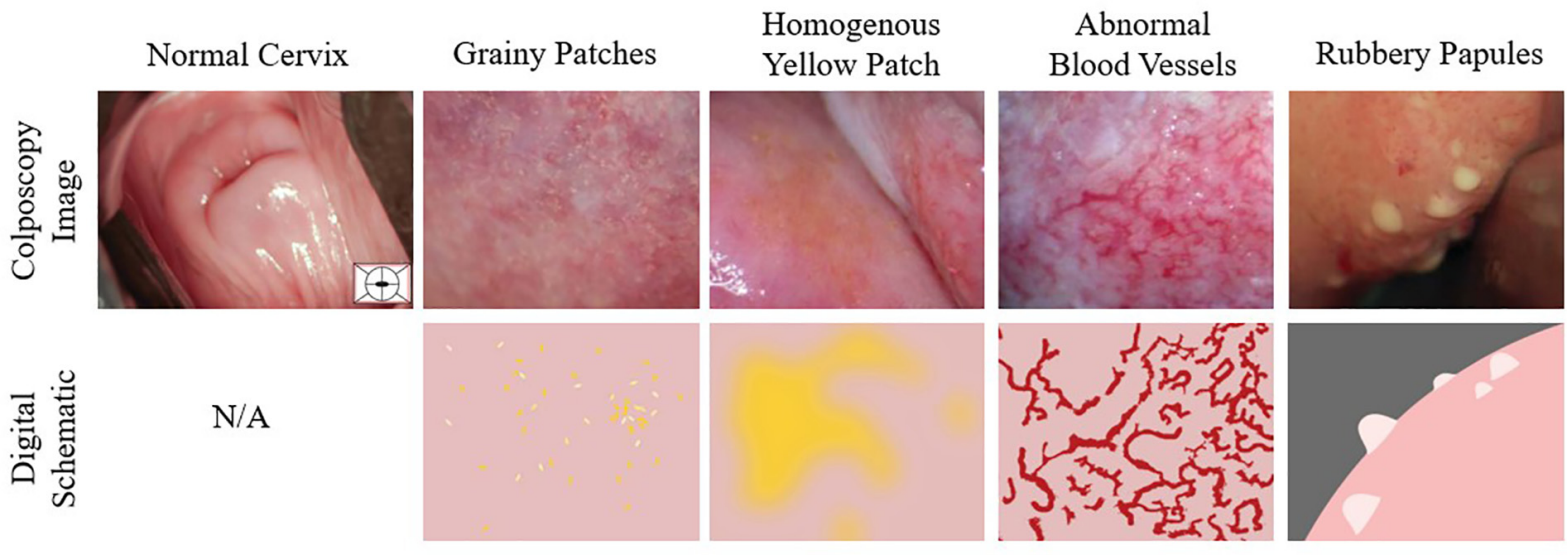
Characteristic lesions of female genital schistosomiasis, showing both colposcopy images and digital renditions of the symptoms. From the World Health Organization’s (WHO) female genital schistosomiases pocket atlas,^4^ with permission. Images taken by: University of KwaZulu-Natal, South Africa and Oslo University Hospital, Norway and Pasteur Institute, Madagascar.

On doing a literature search, we found only 2 published tools for computerized image analysis for FGS,^10^, ^11^ which were both developed by the same research group. One algorithm relied on colorimetric analysis,^10^ whereas the other focused on characterizing the patterns of the blood vessels present in symptomatic patients.^11^ Neither study used modern machine learning methods, which leaves room for the development of new machine learning–based FGS-specific diagnostic algorithms.

In recent years, there has been marked focus on the development of automated computer-aided image diagnostic (CAD) tools for cervical cancer. Both FGS and cervical cancer follow similar screening procedures of performing a colposcopy examination and identifying abnormal lesions on the cervix, but cervical cancer is studied much more prevalently. Therefore, in this study, we aimed to examine the tools that have been developed for cervical cancer and review their applicability to FGS diagnosis.

Softeland et al^12^ performed a systematic review of handheld colposcope hardware devices used for cervical cancer screening that could also be used for FGS screening. However, a sufficient image-capture hardware device is only one component of an effective, automated, single-point-of-care diagnostic tool; the other need is for automated diagnostic image analysis algorithms that can make an accurate prediction. Therefore, in this article, we examined the published literature on automated image diagnosis algorithms for cervical cancer that use colposcopy images and evaluated the feasibility of applying these methods to an automated FGS CAD tool.

## Methods

### Search Strategy and Selection Criteria

We systematically searched the published literature in 2 databases accessed by OVID from the database inception to June 10, 2022: Embase (from 1974) and MEDLINE (from 1946). Two authors (E.J. and M.G.) adapted the keywords used by Softeland et al,^12^ to generate the following search terms: (cervix uteri OR uterine cervical dysplasia OR cervical atypia OR cervical precancer OR cervix OR cervical cancer) AND (colposcop∗ OR digital colposcop∗ OR smartphone∗ OR cervicograph∗ OR photo colposcop∗ OR cervigram∗ OR computerized colposcop∗ OR magnifying OR lightsource OR Gynocular OR EVA System OR mobile ODT OR mobile colposcop∗ OR pocket colposcop∗ OR mobile health OR mHealth OR mobile medic∗ OR mMedic∗ OR equipment design OR mobile phone) AND (comput∗ OR algorith∗ OR automat∗ OR learning) AND (image OR photo∗ OR picture OR cervicograph∗ OR photo colposcop∗ OR cervigram∗).

Identified references were first deduplicated within the OVID database portal and exported to Microsoft Excel 2016 for screening by a single reviewer (E.J.). Editorials, letters, and notes were immediately excluded, but all other reference types and target populations present in the search results were considered. References were first screened by titles and abstracts, and only the relevant articles were followed by full-text extraction. On performing full-text retrieval, records that only included conference abstracts were also excluded. No restrictions were placed on language or date of publication, but all relevant records found were in English and published since 2012.

Diagnostic imaging algorithms that included a likelihood of disease based on the detection of abnormalities in cervical images and image segmentation algorithms that focused on identifying the location of lesions within a cervical screening image were included in this review. However, diagnostic imaging studies that relied primarily on Papanicolaou smear data and histopathologic slides for cervical cancer were excluded.

We also excluded studies that developed image analysis methods for temporal or color-change imaging data. Standard cervical cancer screening procedures such as visual inspection with acetic acid (VIA) and visual inspection with Lugol’s iodine (VILI) change the color of the cervix once the solution is applied, making any lesions present more visible and easier to identify.^13^ It can take up to 2 minutes for these changes to reach maximum intensity on the cervix,^14^ which is why several cervical screening algorithms use a video or time-lapsed images to help identify changes in the appearance of the lesion and its surrounding over time.

Another common practice is for healthcare workers to examine the native cervix under a green or blue light filter.^15^ Blood is red in color and, therefore, will absorb green and blue light. Consequently, areas with visible blood vessels will appear darker in these color-adjusted images, and atypical blood vessels are easier to identify. Hence, a cervical cancer diagnostic algorithm may use both images of the native cervix and these color-adjusted images as input data to guide diagnostic prediction.

We define both aforementioned approaches as multistate imaging because the cervix is represented in multiple different states (either in time or in color). Because multistate imaging may negate the often-characteristic yellow discoloration of FGS lesions by using green or blue filters, they are deemed irrelevant for FGS diagnosis. FGS is typically diagnosed by healthcare workers examining the native cervix in a single state, without relying on any sort of temporal or color changes. Following this definition, cervical cancer algorithms that only use images taken after the application of acetic acid at a single state point in time were included in our analysis.

### Data Analyses

A single reviewer (E.J.) extracted data on the characteristics and performance of the different approaches using a custom spreadsheet. We relied solely on data provided in the published works when performing our analysis. Several data points were collected for each study, such as information about the computational algorithm used, classification performance metrics, and data used to train and test the algorithm. The information about data class breakdowns was based on the number of original images in the data sets used, and excluded any data augmentation that may have been performed as part of machine learning training for instance.

The algorithms were further scrutinized to highlight any design features that could also be relevant to an FGS-specific CAD. These included characteristics such as performing color analysis, texture analysis, and image preprocessing to isolate the cervix, or region of interest (ROI), within the image before running the image through the model. Texture analysis includes the identification of abnormal textures on the cervical tissue and the morphologic patterns of abnormal blood vessels on the cervix. These features were selected because they were used in the 2 currently published tools for computational FGS diagnosis.^10^, ^11^

Additional investigation of the studies was performed to examine the interpretability and public accessibility of the software. Interpretability encompasses characteristics such as whether the algorithm is custom designed from scratch or a standard solution and whether the algorithm identifies where the lesions are present on the image (lesion detection/localization), rather than just provide an image-level classification. Public accessibility is defined as whether the authors provide public access to their code.

For the reviewed cervical screening algorithms, we included sensitivity, specificity, and accuracy^16^ as the relevant performance metrics for the reviewed cervical screening algorithms. These are calculated based on the true-positive (TP), true-negative (TN), false-positive (FP), and false-negative (FN) rates generated by the algorithm. Equations for sensitivity, specificity, and accuracy are outlined in the following equations:

$$Sensitivity=\frac{TP}{TP+FN}$$

$$Specificity=\frac{TN}{TN+FP}$$

$$Accuracy=\frac{TP+TN}{TP+TN+FP+FN}$$

Some studies provided additional performance metrics or used alternative methods of evaluating algorithm performance, but these figures were excluded owing to inconsistencies in reporting across the studies. If multiple algorithms were tested in a study, the one with the best sensitivity was deemed most relevant for selection for an FGS screening tool. This is because, for an FGS screening tool, we want the diagnostic algorithm to be as sensitive as possible to help identify any possible abnormalities for reasons outlined in the discussion section.

If the data splitting ratio for training and testing data was not explicitly provided by the authors, we manually computed the number using the data breakdowns reported in the text. Studies that used cross-validation reported their evaluation metrics as an average of all folds, where in each iteration, one fold is held out from training. This means that they did not use an entirely separate testing set when evaluating their algorithm.

## Results

**Figure 3:**
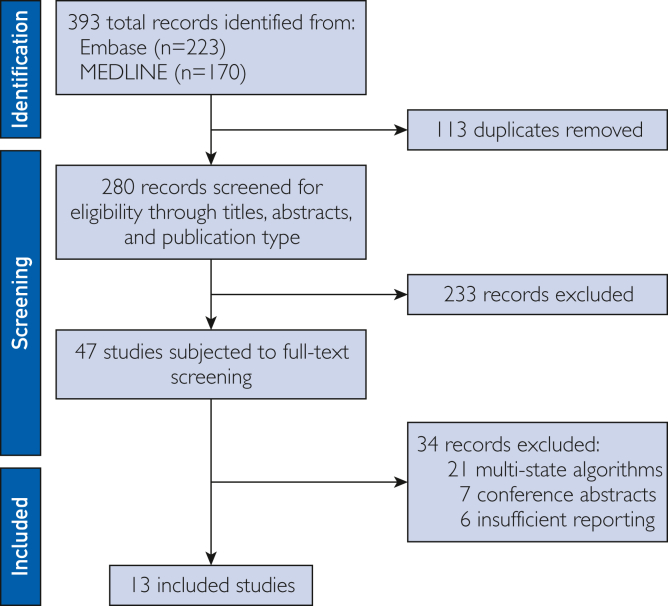
Preferred reporting items for systematic reviews and meta-analyses (PRISMA) flow diagram detailing the selection of studies included in this review.

Our search identified 393 records (Figure 3). We removed 113 duplicates and excluded 233 records after screening for titles, abstracts, and record type. Of the remaining 47 records that were reviewed, 21 studies were excluded because of the main algorithm using multistate techniques that are irrelevant for FGS diagnosis. Furthermore, 7 records were conference abstracts with unavailable full texts. Finally, 6 studies were excluded because of insufficient reporting. Studies with insufficient reporting provided either no description of the data used or data split or no description of the computational algorithm applied. After completing the screening process, 13 studies were included in our literature review.

Cervical cancer uses a cervical intraepithelial neoplasia (CIN) grading system that describes the abnormal changes of the cells that line the cervix. CIN is not cancer, but it is considered a precancerous condition and may require treatment to stop the cervical cancer from developing. The different grades on this scale in the order of increasing severity are normal, CIN1, CIN2, CIN3, and cancer. Most cervical cancer diagnostic algorithms will treat normal and CIN1 images as negative for cervical cancer abnormalities and CIN2, CIN3, and cancer as positive cases for classification. The algorithms in Table 1 that used a multiclass classifier to predict each of the 5 different classes had their data and results recalculated based on the data breakdowns and confusion matrices provided.^17^, ^18^, ^19^, ^20^, ^21^, ^22^, ^23^, ^24^, ^25^, ^26^, ^27^, ^28^, ^29^ In addition, all algorithms listed in Table 1 were either initially presented as binary classifiers (positive or negative for cervical cancer) or recomputed as binary classifiers given the data provided in the study.

**Table 1:** Summary of Eligible Cervical Cancer CAD Algorithms^a^^,^^b^^,^^c^^,^^d^

Reference, year	SEN	SPEC	ACC	Core algorithm	Data class breakdowns	Train: test data split	Data source
Elakkiya et al,^17^ 2021	99.49	96.92	98.55	Faster R-CNN + GAN	1,112 positives; 1,993 negatives	80:20	Kaggle challenge, clinical study
Kudva et al,^18^ 2018	99.05	97.16	97.94	SVM	42 positives; 60 negatives	90-fold CV	Clinical study
Elayaraja and Suganthi,^19^ 2018	97.42	99.36	98.29	Neural Network	—	2-fold CV	NCI Guanacaste dataset
Hu et al,^20^ 2019	82.1^a^ 97.7^b^ 92.9^c^	77.2^a^ 84.0^b^ 83.2^c^	—	Faster R-CNN	228 positives; 8,689 negatives	70:30	NCI Guanacaste dataset
Park et al,^21^ 2021	89.23	93.2	91.14	ResNet50	2,135 positives; 1,984 negatives	80:20	Clinical study
Bai et al,^22^ 2020	85.56	—	—	SE-CNN	3,299 positives; 3,250 negatives	78:22	Clinical study
Bae et al,^23^ 2020	84.1	71.9	80.8	k-NN	15 positives^d^; 5 negatives^d^	50:50	Clinical study
Liu et al,^24^ 2021	82.31	80.04	80.68	ResNet50	4,927 positives; 10,349 negatives	80:20	Clinical study
Li et al,^25^ 2021	81.36	—	81.07	Neural Network	3,894 positives; 4,710 negatives	75:25	Clinical study
Xu et al,^26^ 2016	80.87	75.94	78.41	CNN	345 positives; 345 negatives	10-fold CV	NCI Guanacaste dataset
Li et al,^27^ 2022	73.68	74.42	74.00	DenseNet161 + Vision Transformer	1,403 positives; 1,109 negatives	96:04	Clinical study
Cho et al,^28^ 2020	66.7	69.9	68.9	ResNet152	565 positives; 226 negatives	85:15	3 clinical studies combined
Zhang et al,^29^ 2020	57.56	78.55	73.08	DenseNet121	4,058 positives; 4,781 negatives	93:07	Clinical Study

CNN, convolutional neural network; CV, cross-validation; GAN, generative adversarial network; k-NN, k-nearest neighbors; NCI, National Cancer Institute at the National Institute of Health in the United States; R-CNN, region-based convolutional neural network; SE-CNN, squeeze excitation convolutional neural network; SVM, support vector machine.

a Metrics for women under the age of 25.

b Metrics for women between the ages of 25 and 49 inclusive.

c Metrics for women older than 50.

d Note that Bae et al,^23^ assigned all CIN1+ images as positive and the rest as negative.

Of the 13 algorithms analyzed, 11 of them use neural network architectures. This is indicative of the current trend and success in using neural network-based algorithms for image classification. Regarding data used to train the algorithms, only 4 of the studies used publicly available data sets (ie, Kaggle challenge data set or NCI Guanacaste data set), whereas the other 9 used privately acquired clinical study data.

Table 2 further outlines the algorithmic features that have been included in the design of the identified CAD solutions.^17^, ^18^, ^19^, ^20^, ^21^, ^22^, ^23^, ^24^, ^25^, ^26^, ^27^, ^28^, ^29^ All algorithms performed some type of image preprocessing by first identifying the immediate ROI, which could help reduce the amount of noise or extraneous information within the image. Four models did this process manually by having a user first examine the images and identify the cervical region, whereas the rest of the 9 algorithms used an automated approach. Two of the models assumed that the cervix will be located in the center of the image and performed a naïve cropping to remove the edges of the image. The other 7 models used a more general approach, allowing the algorithm to first identify an ROI within the image. Only 3 algorithms considered color analysis, and only 5 of them performed texture analysis on the image. Because the existing computational methods for FGS diagnosis uses colorimetric and textural features, these specific feature engineering choices could be indicative of the algorithm’s applicability to FGS diagnosis.

**Table 2:** Additional Model Features Included That May be Applicable When Designing an FGS Diagnostic Algorithm

Reference, year	Color analysis	Texture analysis	ROI detection	Algorithm design	Detects lesion location	Open-source code
Elakkiya et al,^17^ 2021			Automatic	Custom		
Kudva et al,^18^ 2018	✔	✔	Automatic	NA		
Elayaraja and Suganthi,^19^ 2018		✔	Automatic	Custom	✔	
Hu et al,^20^ 2019			Automatic	Standard		
Park et al,^21^ 2021			Naïve Automated	Standard	✔	
Bai et al,^22^ 2020			Automatic	Standard	✔	
Bae et al,^23^ 2020	✔	✔	Automatic	NA	✔	
Liu et al,^24^ 2021			Manual	Standard		
Li et al,^25^ 2021		✔	Manual	Custom		
Xu et al,^26^ 2016	✔	✔	Automatic	Custom		Mentioned
Li et al,^27^ 2022			Manual	Custom		
Cho et al,^28^ 2020			Naïve Automated	Standard	✔	
Zhang et al,^29^ 2020			Manual	Standard		

NA, not applicable.

Among the neural network-based architecture designs used, 5 of the 11 used standard neural network models without any custom feature engineering or model design. Only neural network architectures in the included studies were analyzed for customized building blocks, inputs, or loss functions because these are not applicable to support vector machine or k-nearest neighbor algorithms. Moreover, only 5 of the 13 algorithms located the lesion as output. None of the methods had the code publicly available; Xu et al^26^ mentioned the desire to make code publicly available but the returned page link was not accessible.

## Discussion

When designing an automated FGS diagnostic imaging algorithm, some of the most important features are high sensitivity performance and the ability to identify the location of the abnormalities on the cervix. Achieving high sensitivity is more important than achieving high specificity to ensure that as many potential FGS-positive patients as possible are identified and not lost to clinical follow-up. The ability to show the patients and providers where the potential abnormalities are on the cervix may encourage clinical follow-up.

Holmen et al^10^ performed FGS image colorimetric analyses on the characteristic yellow sandy patches, which achieved a sensitivity score of 83%. Using this result as a reference we discuss only the cervical screening algorithms that have a sensitivity score of >83%. Kudva et al^18^ and Bae et al^23^ used significantly smaller data sets, but they both performed specific feature engineering around color and texture analysis. It is also worth noting that these methods did not use a neural network architecture, with Kudva et al^18^ using a support vector machine algorithm and Bae et al^23^ using the k-nearest neighbor algorithm. It may be worth applying their methods on larger data sets to see whether their algorithms achieve similar performance on more data, particularly because Bae et al^23^ used images acquired using a smartphone, which could be directly applicable to the low-resource settings where FGS is endemic.

Elakkiya et al^17^ presented an algorithm with the highest sensitivity score, but their architecture contains a generative adversarial network that is extremely resource intensive from a computational standpoint. It can also be difficult to train a stable generative adversarial network model because the architecture is inherently unstable with the simultaneous training of the component generator and discriminator models.

The algorithm with the next highest sensitivity is the one presented by Elayaraja et al.^19^ This is a promising bespoke algorithm that takes texture analysis into account and is able to identify the location of lesions in the results. However, the authors do not report how much data they used for training or the breakdown of images into the different data classes. In addition, their reported metrics are based on performing 2-fold cross-validation instead of having an entirely separate test set for evaluation. Therefore, the performance metrics may be slightly inflated depending on how the data are used in their study.

The faster region-based convolutional neural network (Faster R-CNN)–based approach presented by Hu et al^20^ has relatively good performance, but it is a preassembled solution, and the algorithm does not detect lesion location. Faster region-based CNN is a single-state model that uses a region proposal network to generate proposed ROIs. This characteristic enables it to save time during training. The same group performed a follow-up study using images acquired using a smartphone and achieved similar results, indicating that this model may be feasible for use in the field.^30^

Park et al^21^ used a standard ResNet50 model, but their study also identified the location of the lesion on the cervix by using a class activation map to show which areas were given more weight in decision-making. Because they used a naïve cropping technique to determine the ROI, the performance of their method could also be increased by applying a more algorithmic approach to image preprocessing to ensure that the ROI is captured within the image being analyzed by the model.

Finally, Bai et al^22^ 2020 used a modified CNN architecture for their model, which included a squeeze excitation block.^31^ The squeeze excitation block is an architectural unit that explicitly models interdependencies among input channels to recalibrate channel-wise feature responses. This helps boost performance because it helps strengthen the signal of important features in an image and suppresses the noise from the nonprimary features in the background. Bai et al^22^ also used an automated ROI selection that likely helped boost performance, and their study detected lesion locations.

On the basis of this review, the models covered in the discussion section are recommended as first-approach candidates for an FGS diagnosis tool. Although some of the methods include CNNs that are resource intensive during training, the application of CNN-based models to test data is relatively quick. Therefore, if a pretrained model could be loaded into a smartphone or tablet application, then it could be used in the field. If these models were to be publicly released, they could be tested on FGS-specific data sets to determine their performance.

The performance of any algorithm will depend on the quality and representativeness of the data provided to train the model. Because FGS is a neglected disease, there are very few data sets available, and none of them are publicly accessible. Moreover, the images that are captured and analyzed largely only depict a view of the cervix, without much of the vaginal wall. If clinicians also rely on the symptoms present on the vaginal wall to make diagnoses, then a computer algorithm will inherently be at a disadvantage with this limited image content. To overcome the limited availability of imaging data, it may be worth adding in other types of data to the model, such as patient clinical data. Liu et al^24^ included clinical data as an additional input to their algorithm, which was shown to boost sensitivity by ∼3 percentage points for detecting cervical cancer. This clinical data could be anything from HPV results to family planning regiments, and further analysis as to the benefits of including these type of data needs to be performed in future studies.

For FGS, there is also an issue of developing a standard for ground truth labels. Currently, labels are determined by expert consensus^2^ or in conjunction with other relevant diagnostic factors; however, these manual diagnoses can be highly variable. Because biopsies have already been ruled out owing to their expensive and invasive nature, one possible solution could be to use molecular diagnostic techniques such as running polymerase chain reactions to screen for *S. haematobium* DNA from vaginal swabs. Several studies have considered the role of molecular diagnosis for FGS.^32^, ^33^, ^34^ Although there are still improvements to be made in accuracy and sensitivity, particularly across different age groups, the consensus is that the polymerase chain reaction could be a viable diagnostic reference method.^35^ A tissue diagnostic standard will in-turn lead to the development of more reliable computational models.

Although several algorithms have been covered in this review and could be applied and tested on FGS diagnosis, there are also limitations in our search. This is because we did not search the reference lists of the texts analyzed, which could have yielded additional relevant studies. In addition, the generation of keywords and search terms may not have been the optimal selection and could have used further iterations. Moreover, the analysis performed in this review was limited owing to the nonstandardized reporting across all studies. This is a well-known challenge in the current image analysis literature, with calls to improve the quality of study reporting.^36^^,^^37^ None of the studies that were covered in this review provided access to their software code, making these studies much more difficult to replicate and use for other applications. Hence, although the 13 identified articles provide valuable insights into CAD techniques that could be applied to FGS, none of these algorithms can currently be directly applied out of the box. In the future, a more standardized data and algorithmic reporting method and code availability should be encouraged for studies that aim to present novel computational solutions for global health imaging problems.

## Potential Competing Interests

Author Jin is partially funded by an Engineering and Physical Sciences Research Council (EPSRC) studentship, and the EPSRC had no influence over this study. Dr Gomes works for Merck KGaA, who also funded author Jin’s travel to the 2022 Geneva Health Forum. Dr Gomes is an employee of Ares Trading S.A., Eysins, Switzerland, an affiliate of Merck KGaA, Darmstadt, Germany. Author Jin and Dr Noble report no conflicts of interest.
